# Astrocytes serve as integrative hubs regulating sleep and arousal

**DOI:** 10.1371/journal.pbio.3003389

**Published:** 2025-10-03

**Authors:** Ashley M. Ingiosi

**Affiliations:** Department of Neuroscience, Ohio State University, Columbus, Ohio, United States of America

## Abstract

Astrocytes are increasingly understood to modulate sleep and arousal. This Primer highlights a recent PLOS Biology study elucidating the mechanisms of this regulation, revealing that histamine-1-receptor signaling in astrocytes modulates intracellular calcium and extracellular adenosine dynamics to regulate arousal.

## Teaser

Astrocytes help control sleep and arousal. A new study in *PLOS Biology* shows that a wake-promoting signal called histamine affects how astrocytes respond to and modulate other brain signals and reduces time spent awake through astroglial histamine-1-receptors.

Astrocytes are non-neuronal cells that make up one of the largest cell populations in the central nervous system, often outnumbering neurons. They play essential roles in maintaining brain function which include providing structural and metabolic support for neurons, regulating blood flow, and responding to immune challenges. Recent work, however, has revealed that astrocytes are more than supportive bystanders—they can directly influence behavior. Some of the earliest *in vivo* evidence emerged about 15 years ago when disrupting astrocyte communication impaired the brain’s ability to balance sleep need with sleep loss in mice [1]. Since then, studies have continued to uncover diverse roles for astrocytes in sleep and wakefulness in various species from invertebrates to mammals, indicating these star-shaped cells are essential for sleep-wake regulation [2,3]. Despite this progress, we are in the early stages of understanding how astrocytes influence behavior, and several fundamental questions remain regarding how astrocytes orchestrate a delicate balance between sleep and wakefulness.

**Fig 1:**
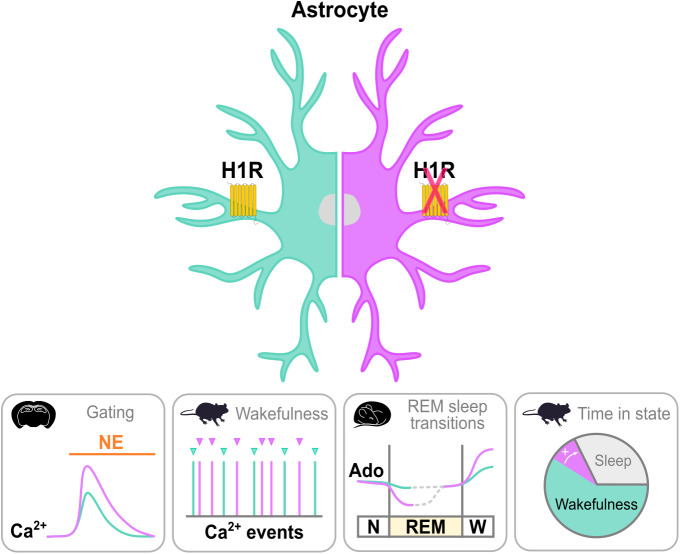
Astroglial H1Rs influence neuromodulatory integration and arousal. How histamine modulates arousal via astrocytes was investigated in brain slices and mice with (left; green) and without (right; purple) histamine-1-receptor (H1R) expression in astrocytes of the primary visual cortex (V1). Panels below depict, from left to right, that H1R expression in V1 astrocytes: (1) attenuates astroglial calcium (Ca²⁺) responses to norepinephrine (NE) in brain slices; (2) suppresses astroglial Ca²⁺ events during wakefulness; (3) restricts the dynamic range of extracellular adenosine (Ado) around rapid eye movement (REM) sleep transitions (N, non-rapid eye movement sleep; W, wakefulness); and (4) reduces time spent in wakefulness.

In this issue of *PLOS Biology*, Taylor and colleagues [4] address one of these fundamental questions: which astroglial signaling pathways mediate arousal? Our understanding of astroglial control of arousal is based primarily on the wake-promoting neuromodulator norepinephrine (NE) [5,6]. But there are several wake-promoting signals in the brain, and astrocytes express receptors for these neuromodulators. In this study, Taylor and colleagues investigated how histamine—another wake-promoting neuromodulator [7]—acts through astrocytes to integrate neuromodulatory inputs and influence arousal using a combination of genetic, pharmacological, and imaging techniques in brain slices and behaving mice (Fig 1).

Starting with two-photon dynamic imaging in brain slices, the authors found that astrocytes in the primary visual cortex (V1) responded to histamine with elevated intracellular calcium—a measure of cellular activity. This response was dependent on astrocyte-specific histamine-1-receptors (H1Rs). H1Rs also attenuated astroglial calcium responses to NE, as deleting H1R selectively in astrocytes increased their responsiveness to NE regardless of prior histaminergic stimulation. These findings suggest that H1Rs mediate astroglial responses to histamine and gate responses to non-histaminergic signals, allowing astrocytes to tune their activity to multiple neuromodulatory inputs. This observation is similar to recent work in fruit fly larvae and cultured rat astrocytes showing NE (and its fly homolog) can gate astrocyte calcium responses to other neurotransmitters and neuromodulators [8].

Astroglial responsiveness to histamine was confirmed *in vivo* via dynamic fluorescence recording using fiber photometry in mice naturally cycling through sleep and wakefulness. In V1, population-level astrocyte calcium activity (e.g., events, amplitude) was higher during electroencephalographic-defined wakefulness compared to non-rapid eye movement (NREM) sleep and rapid eye movement (REM) sleep, consistent with state-dependent changes in histamine levels and astroglial calcium changes in other cortical areas [2]. Mice lacking H1R expression in V1 astrocytes exhibited elevated astrocyte calcium levels during transitions from NREM sleep to wakefulness and more calcium events during wakefulness. This result was unexpected given that deleting astroglial H1Rs abolished calcium responses to histamine in brain slices. However, this finding aligns with slice data showing that H1R deletion increased astroglial responses to NE, which is also at high levels during wakefulness. *In vivo* studies will be needed to determine if non-histaminergic neuromodulators contribute to the observed calcium response in mice lacking V1 astroglial H1Rs.

The authors next investigated whether histaminergic signaling through astrocytes influences extracellular levels of adenosine—a sleep-promoting neuromodulator released by astrocytes to alter neuronal activity [9]. As expected, adenosine was higher during wakefulness relative to NREM and REM sleep. However, deleting H1R in V1 astrocytes increased the dynamic range of extracellular adenosine around REM sleep transitions, a period when histamine levels are low. One interpretation offered is that the impacts of histaminergic H1R activation during wakefulness persist into sleep. Alternatively, H1Rs may be constitutively active during REM sleep as brain slice experiments showed H1R can modulate NE-induced astroglial calcium activity even in the absence of histaminergic stimulation.

Sleep phenotyping studies revealed that deleting H1Rs unilaterally in V1 astrocytes increased time spent in wakefulness and reduced time in REM sleep during the latter half of the light period. Given that histamine promotes wakefulness through neuronal pathways, this result was unexpected. However, H1R is a G_q_-coupled receptor, and this finding aligns with previous work from this research group showing that G_q_-signaling in V1 astrocytes mediates NREM sleep duration [10]. Together, these results suggest that H1R serves different functional roles in astrocytes and neurons.

Through this compelling work, the authors demonstrate that astrocytes can integrate and tune simultaneous inputs from distinct neuromodulators via H1R. This raises an important question: is this integrative function unique to histamine and H1R, or can it also occur through other G-protein-coupled receptors or signaling pathways? Studies in larval fruit flies show NE, but not all neurotransmitters, can similarly gate astroglial responses to neuromodulators [8].

The authors also showed that astroglial H1R expression modulates extracellular adenosine levels around REM sleep transitions. Astroglial regulation of sleep need has been linked to astrocyte release of adenosine triphosphate (ATP)—which is quickly hydrolyzed to adenosine—since the initial *in vivo* study of astroglial sleep-wake regulation [1]. However, the precise mechanisms of ATP/adenosine release—particularly vesicular exocytosis—is a topic of ongoing debate [11,12] with key concerns centered on cellular specificity and physiological relevance of experimental approaches [11]. A recent study using a newer technique to optogenetically stimulate astroglial G_q_-signaling in somatosensory cortex of anesthetized mice reported that ATP is released from astrocytes via vesicular exocytosis in a calcium-dependent manner [13]. ATP release events had a median duration of ~30 s and emerged after 2–3 min of sustained stimulation. These findings lend support for astroglial regulation of brain processes that unfold over prolonged timescales, like the accumulation and discharge of sleep need. Continued investigations into astrocyte-specific mechanisms of gliotransmitter release will be essential for understanding how astrocytes regulate sleep–wake expression and sleep need as well as their broader influence on neuronal activity, neural physiology, and behavior.

Another question raised is whether the physiological and behavioral effects observed by Taylor and colleagues extend to the dark period. Assessments were conducted during the latter half of the light period, which is the mouse’s rest phase. Evaluating the impact of impaired histaminergic signaling in astrocytes during the dark period—when mice spend more time awake—may offer additional insights into astroglial control of arousal. Although deleting astroglial H1Rs throughout the brain does not change time spent in sleep and wakefulness across the 24-h day [14], it is important to investigate region-specific impacts of astrocytes which are a molecularly and functionally diverse cell population. Related, does histaminergic gating of NE vary with sleep need? If so, one might predict that H1R-mediated attenuation of wake-promoting NE will be stronger when sleep need is high (e.g., early in the light period) and weaker when it is low (e.g., early in the dark period).

Overall, Taylor and colleagues highlight a growing role for astrocytes in the control of arousal, presenting new evidence that astrocytes can integrate signals from multiple wake-promoting neuromodulators in a way that can influence physiology and behavior. Future work should aim to uncover the intracellular mechanisms underlying this neuromodulatory integration and downstream signaling in astrocytes. Additionally, since many signaling pathways converge on calcium, it will be important to determine how astrocytes decode calcium dynamics to generate distinct physiological and functional outcomes.

## Abbreviations

ATP: adenosine triphosphate
H1Rs: histamine-1-receptors
NE: norepinephrine
NREM: non-rapid eye movement
REM: rapid eye movement
V1: primary visual cortex
